# Acute‐on‐chronic food protein‐induced enterocolitis syndrome in an exclusively breast‐fed infant

**DOI:** 10.1002/ccr3.1905

**Published:** 2018-11-13

**Authors:** Maria Ntoumpara, Fotini Sotiriadou, Maria Fotoulaki

**Affiliations:** ^1^ 4th Department of Pediatrics Aristotle University of Thessaloniki Thessaloniki Greece

**Keywords:** acute‐on‐chronic FPIES, breast‐fed, food protein‐induced enterocolitis syndrome

## Abstract

Food protein‐induced enterocolitis syndrome (FPIES) is a non‐IgE‐mediated gastrointestinal food hypersensitivity disorder, typically provoked by cow's milk or soy in formula‐fed infants. This case shows that diagnosis of FPIES should be suspected in exclusively breast‐fed infants and pediatricians should be suspicious of this in infants with shock and sepsis.

## 1 INTRODUCTION

Food protein‐induced enterocolitis syndrome (FPIES) is a non‐IgE‐mediated gastrointestinal food allergy expressed with the acute or chronic phenotype.^1^ The acute form is the commonest type and is characterized by repetitive vomiting, lethargy with pallor, ashen appearance, and diarrhea within 2‐6 hours from the induction of food. Hypothermia, elevated peripheral blood neutrophil, and thrombocytosis have also been reported. Chronic FPIES presents intermittent emesis, watery diarrhea, dehydration, acidemia, failure to thrive, and weight loss. The age of onset seems to be influenced by the time of direct introduction of food proteins in infants’ diet.^2^ Cow's milk (CM) and soy proteins are frequently the triggers, whereas solid foods like rice, oats, barley, peas, poultry, or any food protein can be a trigger.^3^, ^4^ The diagnosis of FPIES is based on the history and clinical symptoms (Powell's criteria and Leonard's newly propped criteria)^4^ after excluding other possible causes. Oral food challenge (OFC) is proposed as the “gold standard”^4^ for the FPIES diagnosis. Due to the rarity of FPIES caused by protein passed through maternal milk, we describe a case of an exclusively breast‐fed infant with acute on chronic FPIES that was initially misdiagnosed as septic patient.

## 2 CLINICAL CASE

An exclusively breast‐fed infant, presented at the age of 4 months to the local hospital emergency department with persistent diarrhea (10‐12 episodes during the last 8 days), not gaining weight from his third month of life, anorexia, protracted vomiting, without fever.

Physical examination revealed pallor, hypotonia, and reduced skin turgor; heart rate, blood pressure, auscultatory cardiac and respiratory findings and neurologic examination were normal. The laboratory investigation revealed: WBC, 5.51 K/μL (NEUT) 20.2% (LYM) 64.4% (MONO) 13.8% (EOS) 0.6% (BASO) 1%; PLT, 450 K/μL; Hb 12.1 g/dL; HCO3, 23.96 mEq/L; base excess, up to −3.3). A gastrointestinal infection was suspected and an intravenous fluid resuscitation therapy was implemented. Hypokalemia, anemia, neutrophilic leukocytosis, thrombocytosis, and metabolic acidosis were observed during the fifth day of his nursing (K+, 2.77 mEq/L; Hb, 9.7 g/dL; WBC, 12.40 K/μL (NEUT) 52%, (LYM) 39.4%, (MONO) 7%, (EOS) 0.6%, (BASO) 1%; PLT 530 K/μL; Hb 9.1 g/dL; HCO3, 14.6 mEq/L; base excess, up to −13). The initial diagnosis of viral gastroenteritis was disputed due to the persistent clinical symptoms, the absence of improvement of his general condition, and the deterioration of laboratory tests. All the aforementioned raised suspicions for possible sepsis. Therefore, antibiotic treatment was admitted which was discontinued when C‐reactive protein was normal, and urine and blood cultures proved negative. Because his family history presented an older sister with cow's milk allergy and due to the worsening of his clinical after the ingestion of 10 mL of partially hydrolyzed formula, IgE levels and RAST tests were also performed in case of cow's milk allergy or other IgE‐mediated gastrointestinal allergy. IgE levels were low, and RAST tests to cow's milk proteins were negative. With the suspicion of FPIES, a restriction in mother's diet was suggested and mother's consumption of milk, dairy products, and soy was eliminated as the most common foods responsible for FPIES in breast‐fed infants. Because this strategy did not bring results during the next ten days, interruption of breast‐feeding was proposed and an amino acid‐based formula was provided for two weeks, leading in a progressive symptoms’ improvement. The reintroduction of breast‐feeding while the mother was on strict elimination diet from the above‐mentioned foods, resulted in the reappearance of diarrhea, anorexia, and weakness after six hours of ingestion. An amino acid‐based formula was issued again avoiding further FPIES episodes. The introduction of the solid foods to the infant‘s diet was performed gradually after the sixth month of age, and cow's milk formula was given after the twelfth month of age under medical supervision. The child has not experienced any further FPIES episodes.

## 3 DISCUSSION

Proteins in breast milk may cause FPIES in exclusively breast‐fed infants, but it is very rare and few cases have been reported so far.^5^, ^6^, ^7^ Cow's milk is the culprit food of the majority of the reported cases of FPIES in breast‐fed infants. The clinical presentation seems to be similar to chronic FPIES.

In our patient, persistent diarrhea, protracted vomiting, and failure to gain weight were the signs of chronic FPIES. The infant was experienced acute FPIES reactions on chronic form with repetitive vomiting, pallor, lethargy, and metabolic acidosis after consuming a small amount of partially hydrolyzed formula. This kind of formula, as complementary to breast milk, was selected based on the family history of cow΄s milk allergy. Chronic FPIES episodes in exclusively breast‐fed infants were described by Tan et al,^5^ Monti et al,^6^ and Sopo et al^7^ Ιn their reported cases, the direct consumption of soy (Tan et al) or cow's milk formula from the exclusively breast‐fed infant was the cause of acute reaction. In our patient, the acute FPIES reaction was observed even with the consumption of partially hydrolyzed formula.

The exclusion of the offending food from the maternal diet remains controversial in cases of acute FPIES episodes caused by the direct ingestion of this food. Järvinen and Nowark‐Wegrzyn^8^ recommended that the culprit food should only be removed if reactions after breast‐feeding have occurred or if the infant fails to thrive. In our case, soy, milk, and dairy products were strictly excluded from the mother's diet.

An interesting also aspect in this case was the persistence of the symptoms even after the maternal strict elimination diet, which were managed only by amino acid‐based formula. This raised the suspicion that FPIES in our patient could be triggered also by other food proteins of human milk. This suspicion was confirmed by the fact that OFC with maternal milk was positive while the mother was on strictly eliminated diet from cow's and soy milk and that was the reason that the introduction of the solid foods to the infant‘s diet after the sixth month of age was performed under medical supervision.

Our initial diagnosis and management of FPIES as sepsis has also been described by Kaya et al^9^ in their reported cases of FPIES in exclusively breast‐fed infants. Diagnosis of FPIES through breast milk is not an easy case, and the exclusion of various alternative illnesses is required.

In conclusion, we hope to contribute to a more suspicious pediatricians’ approach in similar cases. Despite the existence of the possibility of FPIES caused by proteins passed through breast milk, we definitely support that breast‐feeding should be encouraged, with the exclusion of the offending food from the maternal diet.

## CONFLICT OF INTEREST

None declared.

## AUTHOR CONTRIBUTION

MN: involved in write‐up and study design. FS: involved in data collection. MF: involved in data collection and literature review.
